# MRI texture analysis parameters of contrast-enhanced T1-weighted images of Crohn’s disease differ according to the presence or absence of histological markers of hypoxia and angiogenesis

**DOI:** 10.1007/s00261-016-0657-3

**Published:** 2016-02-11

**Authors:** Gauraang Bhatnagar, Jesica Makanyanga, Balaji Ganeshan, Ashley Groves, Manuel Rodriguez-Justo, Steve Halligan, Stuart A. Taylor

**Affiliations:** Centre for Medical Imaging, University College London, 250 Euston Road, London, NW1 2BU UK; Institute of Nuclear Medicine, University College London, London, UK; Histopathology Department, University College London Hospitals, London, UK

**Keywords:** MR enterography, Crohn’s disease, Texture analysis, Angiogenesis, Hypoxia

## Abstract

**Purpose:**

To investigate if texture analysis parameters of contrast-enhanced MRI differ according to the presence of histological markers of hypoxia and angiogenesis in Crohn’s disease (CD).

**Methods:**

Seven CD patients (mean age 38 (19–75), 3 male)) undergoing ileal resection underwent 3T MR enterography including axial ultrafast spoiled gradient-echo T1 post IV gadolinium chelate. Regions of interest were placed in bowel destined for resection and registered to trans-mural histological sections (*n* = 28 across 7 bowel sections) via MRI of the resected specimen. Microvessel density (MVD) and staining for markers of hypoxia (HIF 1α) and angiogenesis (VEGF) were performed. Texture analysis features were derived utilizing an image filtration-histogram technique at spatial scaling factor (SSF) 0–6 mm, including mean, standard deviation, mean of positive pixels, entropy, kurtosis and skewness and compared according to the presence or absence of histological markers of hypoxia/angiogenesis using Mann–Whitney *U*/Kruskal–Wallis tests and with the log of MVD using simple linear regression.

**Results:**

Mean, standard deviation and mean of positive pixels were significantly lower in sections expressing VEGF. For example at SSF 6 mm, median (inter-quartile range) of mean, standard deviation and mean of positive pixels in those with VEGF expression were 150.1 (134.7), 132.4 (49.2) and 184.0 (91.4) vs. 362.5 (150.2), 216.3 (100.1) and 416.6 (80.0) in those without (*p* = 0.001, *p* = 0.004 and *p* = 0.001), respectively. There was a significant association between skewness and MVD (ratio 1.97 (1.15–3.41)) at SSF = 2 mm.

**Conclusions:**

Contrast-enhanced MRI texture analysis features significantly differ according to the presence or absence of histological markers of hypoxia and angiogenesis in CD.

Abnormally increased neoangiogenesis is a histopathological hallmark of Crohn’s disease (CD) [1–3]. Increased hypoxia-inducible factor 1α (HIF 1α) and vascular endothelial growth factor (VEGF) expression is frequently present in Crohn’s affected bowel [1, 4, 5], along with increased mucosal and submucosal microvessel density (MVD) [2]. While the drivers of neoangiogenesis are complex, an increasing body of evidence suggests that chronic inflammation is angiogenesis dependent [1]. This vasculopathy has been targeted by new therapeutics such as Natalizumab, an a4-integrin blocking monoclonal antibody, recently approved to treat CD [6–8]. Non-invasive imaging methods that quantify angiogenesis in CD could therefore have immediate diagnostic utility, which could guide therapy.

Magnetic resonance enterography (MRE) is used frequently to assess CD. Disease activity is associated with increased mural thickness [9–11] and T2 signal intensity [11, 12], but also with increased contrast-enhanced mural signal intensity [9–12]. Contrast enhancement kinetics in CD are however complex and related to both MVD and disease chronicity [12–14] as well as inflammation. It is intuitive therefore that the mural signal pattern following IV contrast administration likely reflects underlying angiogenesis within the bowel wall. Interrogating this signal beyond simple mean intensity could therefore provide new insights regarding the underlying vasculopathy in CD.

Textural analysis (TA) is a post-processing technique that can be applied to cross-sectional data to facilitate analysis of heterogeneity within selected image regions [15]. The filtration-histogram technique is a commonly employed approach whereby image filtration extracts features of different sizes which allows the histogram distribution of grey-scale levels and/or pixel intensity on computed tomography (CT) and magnetic resonance imaging (MRI) to be quantified subsequently [16]. These features may reflect underlying tissue structure, at least in part. TA has been applied successfully to cross-sectional imaging of cancer [17], particularly to investigate underlying tumour vascularity. For example, Ganeshan et al. demonstrated that TA parameters derived from contrast-enhanced CT images may be imaging biomarkers for tumour hypoxia and angiogenesis in non-small-cell lung cancer (NSCLC) [18]. It is therefore possible that TA of contrast-enhanced MRI images could phenotype CD vasculopathy.

The purpose of this prospective study was to investigate any significant differences between metrics obtained by texture analyses of contrast-enhanced MRE sequences (MRTA) in adult patients with small bowel CD according to the presence of histological markers of hypoxia and angiogenesis obtained from the same location.

## Materials and methods

### Study population

The local research ethics committee approved this prospective study and written informed consent was obtained from all participants. Between January 2012 and September 2014, consecutive patients with proven CD (based on standard clinical, endoscopic and histological criteria) and scheduled to undergo surgical resection of diseased small bowel within 3 months were invited to undergo pre-operative MRE.

Consenting patients were excluded from the current study if they underwent MRI on an MRI scanner other than the specific 3 Tesla (T) machine earmarked for this study (see below) (*n* = 6), were under 16 years of age (*n* = 0), were pregnant (*n* = 0), had contraindications to MRI (such as severe claustrophobia; *n* = 1), or had a pacemaker/metallic implant (*n* = 0). Patients were also excluded if post contrast T1 axial sequences were not performed (*n* = 2) or if this sequence was affected by significant artefact precluding further analysis (*n* = 1). For this initial pilot study, patients scanned on a 1.5 T platform were excluded as post contrast images were non-isotropic (unlike at 3 T) which could affect textural analysis. Furthermore, we wanted to avoid the potential confounder of magnet strength on post contrast enhancement T1 signal. Similarly for this pilot, patients were included if the time between MRI and surgery was greater than 3 months due to unforeseen delays in surgical intervention.

Overall 7 patients were eligible (mean age 36 years, 4F) for this study. Indications for elective small bowel resection included obstructing ileal stricture (*n* = 4), entero-enteric fistulation (*n* = 2) and entero-cutaneous fistula (*n* = 1). The mean temporal interval between MRI and surgery was 55 days (range 5–175).

The Montreal classification and C-reactive protein (CRP) were recorded for each patient up to 5 days prior to surgery.

### MRI imaging protocol

After a 4-h fast, patients ingested 1 L of 2% mannitol solution over 40 min and were scanned in the prone position on a 3 T static magnet (Phillips Achieva 3.2.1.1, Philips Achieva, Philips Healthcare, Best, The Netherlands) using the manufacturer’s body and spine array coils following IV administration of 20 mg hyoscine butylbromide (Buscopan, Boeringer-Ingelheim, Ingelheim, Germany).

The MRI sequence protocol is given in Table 1 and included standard axial and coronal balanced turbo field-echo (BTFE) and half-Fourier acquisition single-shot turbo spin echo (HASTE) images. In addition, a dynamic contrast-enhanced protocol was acquired using an ultrafast gradient-echo T1 high-resolution isotropic volume excitation (THRIVE) sequence. Specifically, following intravenous administration of 18 mls gadopentetate dimeglumine (Magnevist; Berlex Laboratories, Wayne, NJ) into an arm vein at 3 mL/s via power injector (Sonic shot GX, Nemoto, Japan), coronal images were acquired through the entire small bowel volume (TR 2.3 ms TE 1.04 ms, image matrix 224 × 224, voxel size 1.8 × 1.8 × 2 mm, 80 slices, flip angle 10°, SENSE factor 4) with 80 measurements every 3.3 s until 264 s. Thereafter, a breath-hold axial image block was acquired (image matrix 576 × 576, voxel size 2 × 2 × 2 mm, 140 slices, flip angle 10°, SENSE factor 4) commencing 300 s following intravenous contrast injection. This delayed axial post contrast sequence was used for texture analysis.

**Table 1:** 3 Tesla (T) magnetic resonance imaging (MRI) parameters

	Coronal/axial balanced steady-state free precision	Coronal/axial balanced steady-state gradient echo with and without fat saturation	Baseline volume interpolated gradient echo	Dynamic contrast-enhanced	Axial post contrast (300 s)
Field of view (mm)	400 × 340 × 198/375 × 295 × 319	400 × 400 × 171/380 × 235 × 344	390 × 390 × 164	400 × 400 × 164	400 × 261 × 280
No. of slices	36/64	34/69	82	80	140
Stacks	1/1	1/1	1	1	1
Repetition time (ms)	1200/1100	1200/1100	2.3	2.3	2.3
Echo time (ms)	80/80	80/80	1.13	1.04	1.04
Image matrix	400/384	528/512	576 × 576	224 × 224	576 × 576
Slice thickness (mm)	5/4	4/4	2	2	2
Averages	1	1	1	1	1
Flip angle	45°/45°	90°/90°	10°	10°	10°

s, seconds; mm, millimetres; ms, milliseconds

### Image histological registration

The technique for matching sites of histological sampling to pre-operative MRI followed that published previously by Punwani et al. [13]. In brief, within 24 h of surgery, a post-operative MRI scan of the resected specimen (pinned to a board in its correct anatomical orientation) was performed using a single-shot turbo spin-echo (SSTSE) sequence in axial and coronal planes (TR 800 ms, TE 86 ms, matrix 256 × 195, slice thickness 4 mm).

The study coordinator, a researcher with 5 years’ experience of small bowel MRI, reviewed the pre-operative MRI images along with the operating surgeon so as to locate the exact segment of bowel resected (using fixed anatomical landmarks such as the ileo-caecal valve (ICV), site of any stricture/fistula etc.) and then chose one to five image sections (median, three) through the resected bowel on the pre-operative MRI for histological correlation. The sections were selected to encompass the range of disease severity with the resected segment, based on conventional MRI parameters, including bowel wall thickness and T2 signal. Subsequently, the coordinator carefully reviewed the resected specimen with the study histopathologist (15 years of experience in GI histopathology) in order to register selected histological sections with reference to the both the corresponding pre-operative MRI and post-operative specimen scan, again with reference to fixed anatomical landmarks [13].

### Region of interest placement

**Fig. 1:**
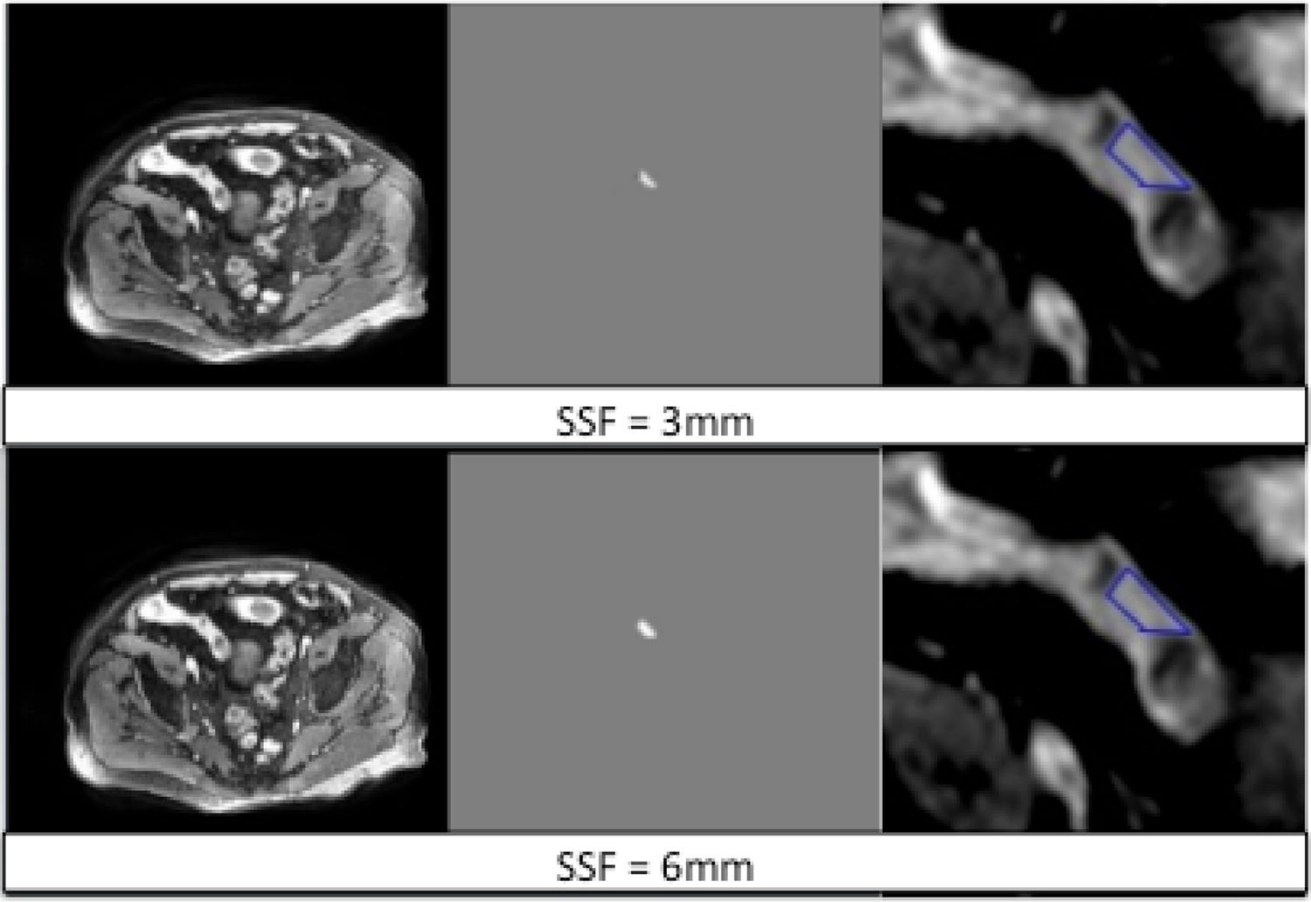
Graphic demonstrating placement of texture region of interest (ROI and TexRAD interface at different specified SSFs. The *top row* reflects the DICOM image analysed (*left*), the image pixels highlighted by the 3 mm SSF (*middle*) and a magnified view of the ROI placed on disease site sampled histologically (*right*). The *bottom row* reflects the DICOM image analysed (*left*), the image pixels highlighted by the 6 mm SSF (*middle*) and a magnified view of the ROI placed on disease site sampled histologically (*right*)

The axial T1 post contrast (THRIVE) weighted images were uploaded into proprietary software for textural analysis (TexRAD, www.texrad.com, part of Feedback Plc, Cambridge, UK) [16]. A radiologist ((GB) with 4 years of experience of MRE) unaware of all clinical and histopathological data (other than the exact site of sectioning) but aware of the study aims placed a single free-hand region of interest (ROI) at each of the pre-identified sections of histological sampling (Fig. 1). The observer reviewed the complete MRE dataset at the time of placing the ROI (OsiriX 64 bit Imaging software, Pixmeo, Geneva, Switzerland) using all available sequences to locate the exact site of histopathological sampling. The ROI was then replicated on the axial T1 post contrast sequence on the textural analysis software, taking care to exclude any luminal or mesenteric tissue but to include the full bowel wall regardless of enhancement pattern.

### Texture analysis

Textural analysis with a filtration-histogram technique was performed within the ROI using previously published methodology [16]. Filtration extracts and enhances texture features apparent at different sizes within ROIs, before subsequent histogram analysis. Specifically, a Laplacian of Gaussian spatial band-pass filter is employed to achieve in-plane filtration, within the ROI. The derived series of images contains features highlighted at different spatial scales, ranging from fine to coarse textures (SSF = 0–6 mm). Histogram quantification then generates the following parameters: mean (average value of the pixels within the ROI), standard deviation (SD, width of the histogram or degree of variation/dispersion from the average), skewness (symmetry of the distribution), mean of positive pixels (MPP, average of the pixel values that are positive), kurtosis (distribution “pointiness” or “sharpness”) and entropy (with increasing irregularity or complexity indicated by a higher entropy value). A simulation study explaining what these texture quantifiers mean in relation to image features is described by Miles et al. [16].

### Histopathological assessment

Histological analysis was performed by a specialist gastrointestinal pathologist (MR) with 15 years of experience, blind to the MRE findings or texture analysis. Immunoperoxidase studies were performed on 4-µm-thick paraffin-embedded sections of formalin-fixed tissue samples. The sections were incubated with antibodies for vascular markers (CD31/CD34), VEGF and HIF1α. Positive immunohistochemical staining for VEGF and HIF1α was noted in epithelial cells/fibroblasts and leukocytes within the bowel wall. In addition to its presence, the intensity of staining in leucocytes only was graded using a 3-point scale: weak, moderate and strong. Microvessels were counted in the most intensely vascularized areas (“hot spots”) at 200× magnification. Mean values for vessel count density (MVD) were calculated as the arithmetic mean of 3 counted hot spots.

### Statistical analysis

The primary analysis was to search for any significant differences between each textural parameter and the presence or absence of histological markers of hypoxia (HIF1 alpha) and angiogenesis (VEGF). A secondary analysis examined for any correlation between microvessel density and textural parameters. All statistical analyses employed SPSS Statistics version 22 (IBM, New York, USA). A sample size calculation was not undertaken. Each textural analysis parameter at each filter level was compared according to the presence or absence of HIF 1α and VEGF in both leucocytes and epithelium/fibroblasts for each histopathological section using Mann–Whitney *U* and Kruskal–Wallis tests as appropriate. To account for multiple comparisons, a *p* value of <0.01 was taken to represent statistical significance.

MVD was measured on a continuous scale. This variable was found to have a positively skewed distribution, and was thus log transformed before linear regression analysis. Results were expressed as ratios that described the relative change in MVD for a given increase in each textural parameter. As there were multiple histological sections from each patient, robust standard errors were employed within the regression analyses to account for data clustering.

Any relationship between histological markers (MVD, HIF and VEGF) was tested using Pearson’s correlation coefficient.

## Results

### Baseline clinical data

Demographic characteristics of the patient cohort are shown in Table 2. The ROIs employed for MRTA contained a mean of 858 pixels (range 247–1964).

**Table 2:** Basic patient characteristics

Patient	Age at diagnosis	Sex (male (M)/female (F))	Montreal classification	Age at surgery	CRP (mg/L)	Medication at time of surgery	Previous surgery	Reason for small bowel resection
P1	16	M	A1L2B3	16	18	Azathioprine	None	Entero-enteric fistula
P2	27	F	A2L1B3	33	72	Azathioprine & infliximab	None	Entero-enteric fistula
P3	71	M	A3L1B2	72	N/P	None	None	Obstructive symptoms due to ileal stricture
P4	10	F	A1L3B1p	17	2	Azathioprine	None	Obstructive symptoms due to ileal stricture
P5	17	F	A2L3B3	43	8	Azathioprine, Infliximab & Budesonide	None	Obstructive symptoms due to ileal stricture
P6	32	F	A2L2B3	45	4	None	None	Entero-cutaneous fistula
P7	9	M	A1L1B2	26	6	Methotrexate & Infliximab	Jejunal and IC resection (2004)	Obstructive symptoms due to ileal stricture

N/P, not performed

### Histological analysis of the microvasculature, hypoxia and angiogenesis

Mean MVD was 42 (19–90). Using a normal upper limit for MVD of 25 [2], 20 of 28 sections (71%) demonstrated abnormal MVD. All 7 patients had a least one section with MVD of greater than 25.

Epithelium/fibroblast VEGF expression was positive in 21 (75%) sections and leucocyte VEGF expression was positive in 13 (46%) sections. Leucocyte VEGF expression was graded as 0 = 15 (54%), 1+ = 0(0%), 2+ = 2 (7%) and 3+ = 11 (39%).

Epithelium/fibroblast HIF 1α expression was positive in 15 (54%) sections and leucocyte HIF 1α expression in 12 (43%) sections. Leucocyte HIF 1α expression was graded as 0 = 16 (57%), 1+ = 3 (10%), 2+ = 2 (7%) and 3+ = 7 (25%).

MVD correlated positively with HIF 1α expression in leucocytes (0.79, *p* = 0.001), VEGF expression in epithelium/fibroblasts (0.52, *p* = 0.005) and VEGF expression in leucocytes (0.73, *p* = 0.001). HIF 1α expression correlated positively with VEGF expression in leucocytes (0.93, *p* = 0.001).

### MRTA parameters differ according to the presence of histological markers of hypoxia and angiogenesis

The complete range of values for all MRTA parameters is shown in Table 3.

**Table 3:** Mean and range of values of magnetic resonance textural analysis parameters

SSF	Mean (range)	SD (range)	Entropy (range)	MPP (range)	Skewness (range)	Kurtosis (range)
0	285.8 (112.0–1102.0)	62.0 (27.4–207.6)	5.0 (4.4–5.7)	285.8 (112.0–1102)	−0.64 (−1.5 to 0.0)	0.2 (−1.2 to 2.1)
2	71.0 (7.6–161.9)	135.7 (64.5–243)	5.7 (5.1–6.3)	139.1 (53.0–241.3)	−0.1 (−0.6 to 0.7)	0.2 (−1.0 to 1.9)
3	119.0 (13.2–269.6)	169.1 (72.8–356.5)	5.8 (5.2–6.4)	192.4 (58.1–364.8)	−0.2 (−0.8 to 0.8)	−0.1 (−0.8 to 2.4)
4	159.3 (19.3–369.5)	181.1 (71.2–491.8)	5.8 (5.2–6.4)	227.5 (58.6–528.1)	−0.3 (−1.1 to 0.6)	−0.1 (−0.9 to 3.1)
5	191.3 (25.0–437.5)	183.7 (72.1–583.8)	5.7 (5.2–6.5)	251.0 (59.9–629.3)	−0.4 (−1.3 to 0.4)	−0.0 (−1.0 to 3.1)
6	216.1 (30.0–487.6)	185.4 (70.4–642)	5.7 (5.1–6.6)	271.0 (62.6–758.5)	−0.5 (−1.3 to 0.6)	0.0 (−1.1 to 3.9)

**Fig. 2:**
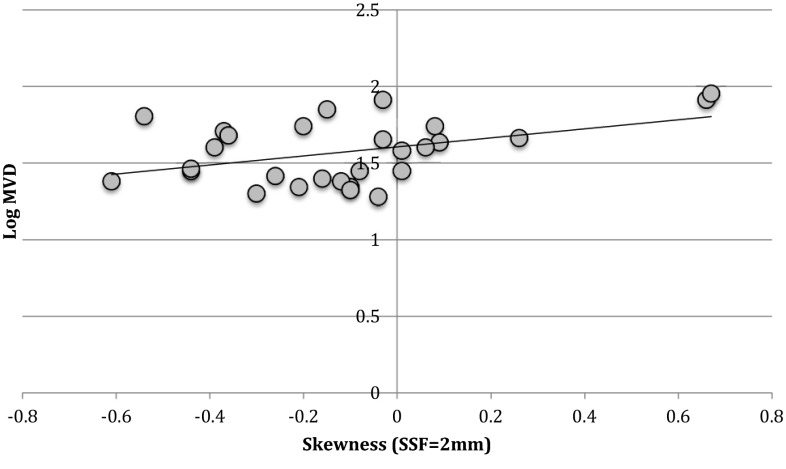
Scatter plot demonstrating significantly positive correlation between skewness and log-transformed microvessel density (MVD) (SSF = 2 mm) (ratio 1.97 (*p* value = 0.014))

Regression analysis revealed a positive association between skewness (SSF = 2 mm) and log-transformed MVD (ratio 1.97 (3.41–1.15) *p* = 0.014) (Fig. 2).

**Fig. 3:**
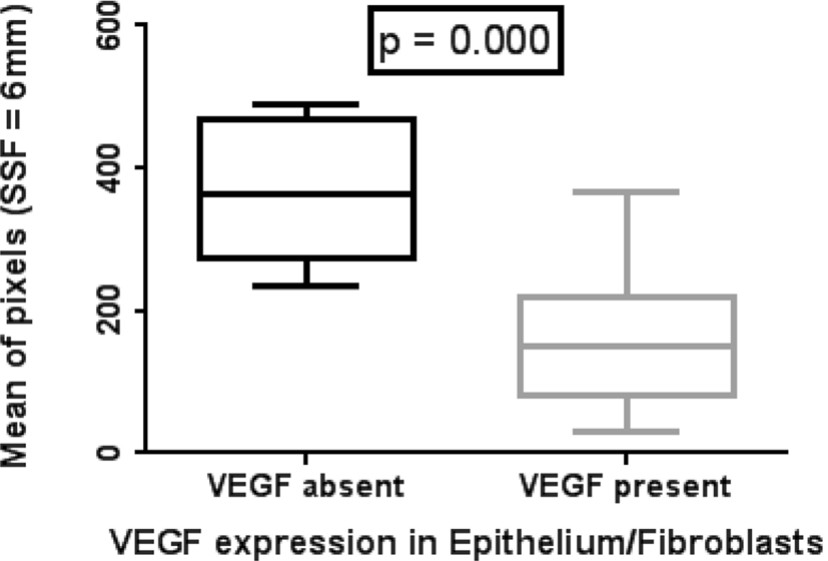
Box-and-whisker plot (minimum, inter-quartile range, median and maximum) (SSF = 6 mm) demonstrating significantly lower mean pixel intensity in sections with vascular endothelial growth factor (VEGF) expression (*n* = 21) in the epithelium/fibroblasts as opposed to those without VEGF expression (*n* = 7)

At the pre-determined level of statistical significance, the mean of filtered pixel intensity was significantly lower in sections expressing VEGF in epithelium/fibroblasts than in those without expression at all filter levels other than SSF = 2 mm (*p* = 0.004 to 0.001) (Table 4; Fig. 3).

**Table 4:** Mean pixel intensity, standard deviation of pixel intensity and mean of positive pixels according to vascular endothelial growth factor (VEGF) expression and filter levels (*Mann–Whitney *p* = 0.01 taken to be significant)

	SSF = 0	SSF = 2	SSF = 3	SSF = 4	SSF = 5	SSF = 6
Mean						
VEGF Present (*n* = 21)	210.5 (85.5)	59.5 (31.1)	96.7 (62.1)	116.2 (75.8)	122.2 (108.0)	150.1 (134.7)
VEGF Absent (*n* = 7)	298.0 (41.4)	100.9 (38.5)	170.3 (50.1)	243.3 (63.5)	306.8 (11.8)	362.5 (150.2)
* p* value*	0.003	0.014	0.004	0.003	0.001	0.001
SD						
VEGF Present (*n* = 21)	45.0 (23.5)	115.7 (50.0)	130.4 (51.7)	139.4 (51.5)	142.4 (42.2)	132.4 (49.2)
VEGF Absent (*n* = 7)	66.8 (39.0)	162.6 (39.9)	220.1 (59.0)	226.2 (56.9)	217.9 (82.3)	216.3 (100.1)
*p* value	0.036	0.023	0.01	0.02	0.012	0.004
MPP						
VEGF Present (*n* = 21)	210.5 (85.5)	118.6 (51.9)	143.6 (58.9)	174.6 (57.4)	181.4 (69.5)	184.0 (91.4)
VEGF Absent (*n* = 7)	298.0 (41.4)	175.3 (17.5)	246.0 (51.6)	296.7 (74.0)	362.7 (150.2)	416.6 (80.0)
* p* value	0.003	0.007	0.003	0.004	0.002	0.001

*n* refers to the number of bowel segments

**Fig. 4:**
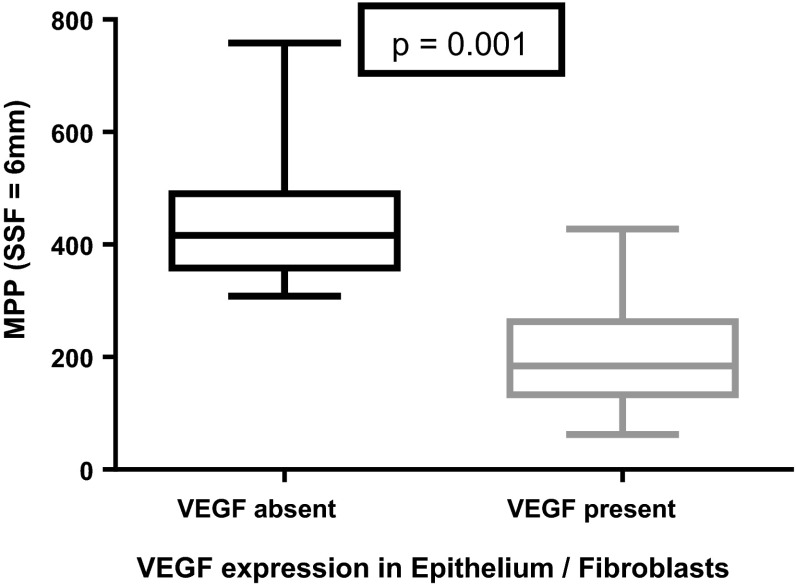
Box-and-whisker plot (minimum, inter-quartile range, median and maximum) (SSF = 3 mm) demonstrating significantly lower mean of positive pixels (MPP) in sections with vascular endothelial growth factor (VEGF) expression (*n* = 21) in the epithelium/fibroblasts as opposed to those without VEGF expression (*n* = 7)

Similarly, MPP was significantly lower in sections expressing VEGF in epithelium/fibroblasts than in those without expression at all filter levels (*p* = 0.007 to 0.001) (Table 4; Fig. 4).

**Fig. 5:**
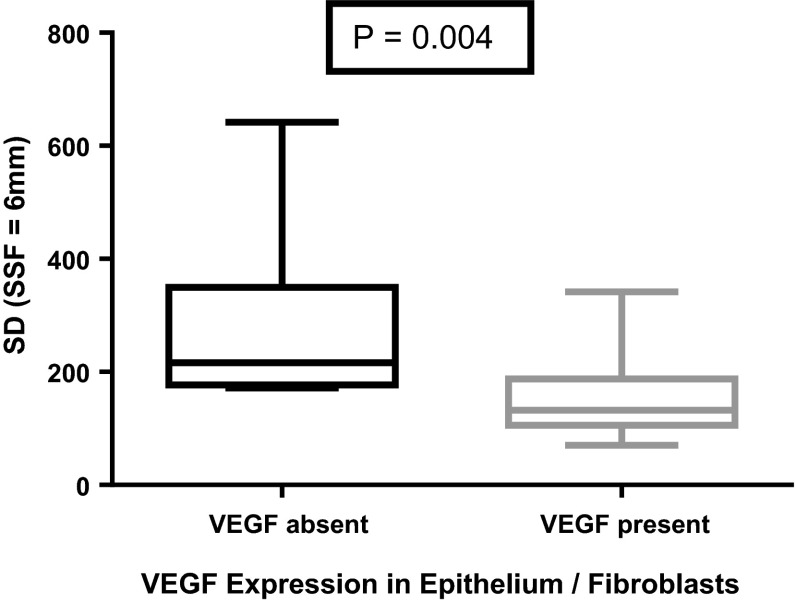
Box-and-whisker plot (minimum, inter-quartile range, median and maximum) (SSF = 6 mm) demonstrating significantly lower standard deviation (SD) of pixel intensity in sections with vascular endothelial growth factor (VEGF) expression (*n* = 21) in the epithelium/fibroblasts as opposed to those without VEGF expression (*n* = 7)

Standard deviation (SD) was significantly lower in sections expressing VEGF in epithelium/fibroblasts at SSF = 3 and 6 (*p* = 0.01, 0.004) (Table 4; Fig. 5).

No other significant differences in MRTA parameters were found according to the presence or absence of HIF 1α or VEGF.

## Discussion

This study demonstrates that several parameters derived from contrast-enhanced MRTA (mean, SD, MPP and skewness) differ significantly according to the presence or absence of histological markers of angiogenesis (MVD and VEGF expression) in CD.

Neoangiogenesis is well described in CD. While inflammation is clearly related to angiogenesis, several alternate processes (such as cell-to-extracellular matrix interaction, vessel wall maturation and basal lamina modifications) are implicated in new vessel development [19]. It is believed that HIF 1α is one of the primary initial stimulants for the pathway [19]. The downstream processes are largely impacted by the ability of leukocytes to release proangiogenic factors such as VEGF and tumour necrosis factor-α (TNF-α) [19]. VEGF in particular is very well characterized as a regulator of angiogenesis and stimulates a sustained angiogenic cascade that is implicated in the pathophysiology of many chronic inflammatory diseases such as rheumatoid arthritis, psoriasis and atherosclerosis [20, 21]. In CD, MVD and VEGF levels are significantly higher in actively inflamed mucosa than in non-inflamed mucosa or mucosa from controls [2].

The current study found that mean (brightness of objects), MPP (brightness of highlighted objects) and SD (variability in the brightness of objects) on post contrast T1 sequences are lower in bowel expressing VEGF in epithelium/fibroblasts compared to bowel without such expression [16]. It would be assumed that tissues with increased vascularity will appear of higher signal on T1 post contrast sequences on MRI. This study also demonstrated a positive correlation between MVD and HIF 1α expression, MVD and VEGF expression, and HIF 1α and VEGF expression. Therefore, we may postulate that hypoxia secondary to reduced blood supply to Crohn’s affected bowel stimulates HIF 1α, thereby increasing VEGF expression, resulting in an increase in the number of microvessels. Work in oncology has demonstrated that neoangiogenic vessels are irregular and function poorly, resulting in inefficient vascular supply that may explain why segments with neoangiogenesis have a reduced and less varied proportion of bright objects [22]. Although there is an association between active inflammation and increased signal intensity on post contrast T1 sequences [23], it is also known that a negative correlation exists between the slope of enhancement and MVD on dynamic contrast-enhanced MRI [24]. It is therefore evident that post contrast mural T1 signal heterogeneity in CD is dependent on the complex interplay between inflammation and angiogenesis.

The findings of the current study parallel similar work using TA in cancer. For example, a negative correlation between SD and MPP, and angiogenic burden (measured by antibodies to CD34) has been demonstrated in NSCLC lung cancer [18]. Miles et al. proposed an algorithm employing a specific threshold of MPP (<17.5) to differentiate between KRAS wild type and mutants, the latter associated with hypoxia and angiogenesis [25, 26]. Our findings are consistent with these studies and indicate that lower values of these texture parameters are associated with the presence of angiogenesis in inflammatory diseases as well as malignancy. Additionally, the findings appear consistent across different imaging modalities such as contrast-enhanced CT and positron emission tomography–CT (PET–CT) [18, 27] adding weight to the assertion that these TA parameters are indeed direct markers of angiogenesis.

We also demonstrated a significantly positive correlation between skewness and MVD (SSF = 2 mm). Positive skewness occurs when there are a larger proportion of bright objects within the ROI and again our findings mirror those from oncology where there is a positive association between skewness (on PET–CT) and angiogenesis in colorectal cancer [27].

In contradistinction to our findings relating MRTA parameters to MVD and VEGF, we found no relationship between MRTA and HIF 1α expression.

Most of our statistically significant observations were evident at multiple SSF levels, especially those >2 mm. Higher SSF levels highlight larger objects within the ROI, which may emphasize more biologically relevant heterogeneity and minimize the impact of intrinsic image noise [16].

Our study has limitations. The sample size is small (necessarily so because the proportion of CD patients who come to resection is small) and, for at least some of the recruits, the temporal interval between MRI and surgery was relatively long. However, our prime aim was to test if the observations linking MRTA parameters to angiogenesis in cancer studies held true for CD. In this regard, this initial exploratory study was successful, although at best our findings are observational. Although using full-thickness histological sections in postsurgical specimens is arguably the strongest reference standard for this type of work, there is a risk of selection bias as, by definition, patients have disease sufficiently advanced and/or complicated to merit surgical resection. ROI placement in normal bowel is technically challenging, but we placed ROIs in thickened abnormal bowel, which is easier. We used previously successful techniques to register imaging and histological sampling sites as exactly as possible. MRTA with TexRad is most reliable when employing isotropic voxels, which limited our analysis to axial T1 post contrast-enhanced images only, which were acquired at 300 s. This is a time point beyond that usually acquired as part of normal clinical practice and may limit the clinical applicability of our findings. However, it could be argued that using such delayed images (when the contrast pool approaches near equilibrium between blood and tissue) our data better reflect intrinsic mural vascularity as opposed to large vessel blood supply (which exerts greater influence at earlier time points post contrast). Furthermore, Rimola et al. have recently described that the use of post contrast imaging at 7 min may help distinguish between fibrosis and inflammation. It would be interesting to test if our observations hold true at this later time point [28]. Like other workers investigating MRTA [29, 30], we did not normalize our post contrast T1 signal intensity values across patients, which could limit inferences regarding mean, SD and MPP values. However, we acquired our data from a single 3T MRI platform with similar gain factor between patients, to mitigate against a lack of normalization. That our findings mirror those published using other imaging platforms such as CT is reassuring that our results are not spurious. As noted above, our data are at best observational and logical next steps would be to investigate TA parameters before and after treatment to see if they could act as biomarkers for therapeutic response.

## Conclusion

Contrast-enhanced MRTA features differ significantly between CD bowel exhibiting histological markers of angiogenesis compared with bowel that does not.

## Abbreviations

CD: Crohn’s disease
TA: Textural analysis
MRTA: MRI texture analysis
MVD: Microvessel density
HIF 1α: Hypoxia-inducible factor 1α
VEGF: Vascular endothelial growth factor
MPP: Mean of positive pixels
SD: Standard deviation
IQR: Inter-quartile range
MRE: Magnetic resonance enterography
CT: Computed tomography
MRI: Magnetic resonance imaging
NSCLC: Non-small-cell lung cancer
T: Tesla
CRP: C-reactive protein
BTFE: Balanced turbo field echo
HASTE: Half-fourier acquisition single-shot turbo spin echo
THRIVE: T1 high-resolution isotropic volume excitation
SSTSE: Single-shot turbo spin echo
ICV: Ileo-caecal valve
ROI: Region of interest
SSF: Spatial scaling factor
TNF-α: Tumour necrosis factor-α
PET–CT: Positron emission tomography–CT
